# An overview on the nutritional and bioactive components of green seaweeds

**DOI:** 10.1186/s43014-023-00132-5

**Published:** 2023-03-20

**Authors:** Jingxiang Xu, Wei Liao, Yuning Liu, Yuling Guo, Shiyue Jiang, Chao Zhao

**Affiliations:** 1 Department of Basic Medicine, Gannan Medical University, Ganzhou, 341000 China; 2 College of Food Science, Fujian Agriculture and Forestry University, Fuzhou, 350002 China; 3 College of Marine Sciences, Fujian Agriculture and Forestry University, No.15 Shangxiadian Road, Fuzhou, 350002 China; 4 Key Laboratory of Marine Biotechnology of Fujian Province, Institute of Oceanology, Fujian Agriculture and Forestry University, Fuzhou, 350002 China

**Keywords:** Green seaweeds, Nutritional components, Bioactivities

## Abstract

Green seaweed, as the most abundant species of macroseaweeds, is an important marine biological resource. It is a rich source of several amino acids, fatty acids, and dietary fibers, as well as polysaccharides, polyphenols, pigments, and other active substances, which have crucial roles in various biological processes such as antioxidant activity, immunoregulation, and anti-inflammatory response. In recent years, attention to marine resources has accelerated the exploration and utilization of green seaweeds for greater economic value. This paper elaborates on the main nutrients and active substances present in different green seaweeds and provides a review of their biological activities and their applications for high-value utilization.

**Graphical abstract:**

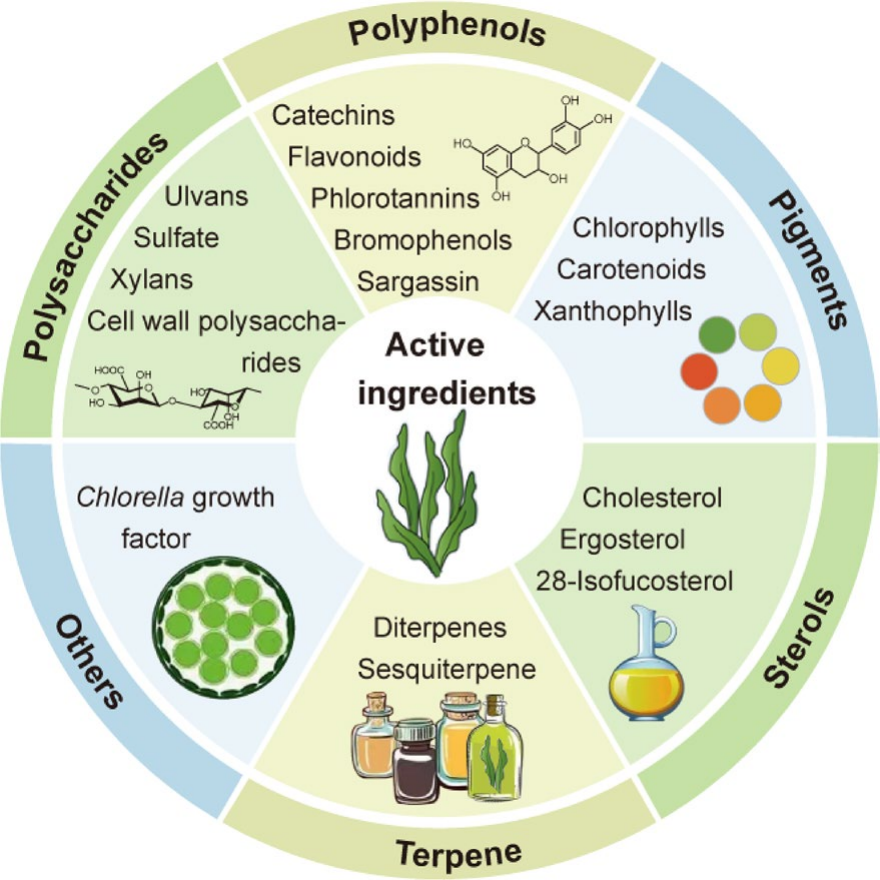

## Introduction

Severe ecological damage causing the desertification of arable land has led to increasing food shortage widely. In the past few years, returning to nature and enjoying green food is becoming an inevitable trend. About two-third of the Earth’s surface is covered with ocean, and the “arable land” area of ocean is about 15 times that of land. The ocean can provide a thousand times more food than all the arable land. Reasonable development and utilization of marine resources will greatly alleviate the problems of food shortage and food security worldwide.

Seaweed is a macroalgae widely found in the ocean, and an important marine biological resource. At present, more than 168,971 seaweed species have been discovered (http://www.algaebase.org), which are taxonomically classified as red seaweed (Rhodophyta), brown seaweed (Phaeophyta), and green seaweed (Chlorophyta), depending on the nature of their pigment abundance (Bleakley & Hayes 2017). Seaweeds have high protein, whereas low-fat content. They are also rich in dietary fiber, vitamins, and minerals (Chan & Matanjun 2017; Rodrigues et al. 2015), which makes them an ideal natural food for consumption. Seaweeds also have high edible and medicinal properties (Hughes et al. 2018; Lozano Muñoz & Díaz 2022; Zhao et al. 2018).

Green seaweeds, as a valuable source of bioactive compounds, are still underutilized in nutraceuticals and pharmaceuticals. They contain several important proteins, polysaccharides, phenolic compounds, etc. (Ibañez & Cifuentes 2013; Kellogg & Lila 2013). For example, *U. prolifera* was characterized as a high-protein, high ratio of unsaturated lipid acids, and low-fat seaweed food (Li et al. 2018). The composition of green seaweeds is affected by species variation, their growth stage, and the environment (Mao et al. 2006; Marinho-Soriano et al. 2006; Verma et al. 2017). The current review focuses on the nutrient compositional differences among different green seaweed species and presents the research progress in the exploitation of green seaweeds and their active substances.

## Description, growth conditions, and distribution

**Fig. 1:**
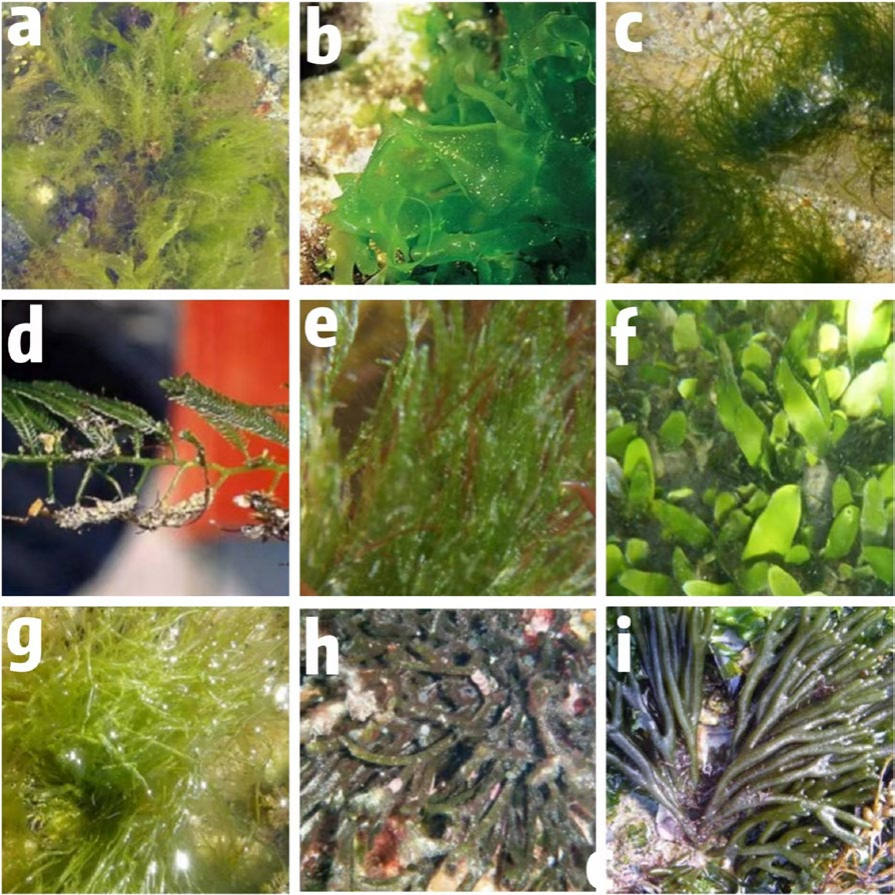
Green seaweeds species and their description, growth, and distribution. **a** *Ulva lactuca* with light yellow-green, dark green color, with irregular and small pores on the leaves and fringed edges, and it can grow to 20–30 cm*;* **b** *U. fasciata* is bright grass green to dark green with a golden color at the edges while reproducing and have thin, lamellar leaf-like bodies consisting of broad blades, 10–15 cm wide at the base, tapering to less than 2.5 cm wide at the tip*;* **d** *Caulerpa taxifolia* is in dark green to light green color, with flattened, feathery leaves*;* **e** *Cladophora prolifera* is usually less than 0.5 mm wide and 3–5 cm long; **f** The leaves of *C. prolifera* are connected by underground stolon, which are long and dense in bright places and thin and long in shady places*;* **g** *Enteromorpha intestinalis* is yellowish green, the seaweeds are tubular, the upper part is swollen to intestinal shape, the lower end is long and pointed, the length can reach up to 35 cm*;* **h** *Codium vermilara* is dark green, large and porous, and morphologically diverse*;* **i** *C. tomentosum* leaves are slender, rounded at the tip, and up to 30 cm long. Pictures are from Portuguese Seaweeds Website: http://www.flordeutopia.pt/macoi/default.php

Green seaweeds are primarily found in the intertidal zone. Common green seaweed species belong to *Ulva*, *Enteromorpha*, *Chaetomorpha*, *Codium,* and *Caulerpa* genera. Four major species of green seaweed belonging to the genus *Ulva* includes *Ulva lactuca, U. prolifera* and *U. linza* (Miao et al. 2020) (Fig. 1a-c & g). *U. lactuca* also known as sea lettuce, is commonly grown on rocks and found naturally in sublittoral waters around the world (Geertz-Hansen et al. 1993; Guiry 2021). It can grow year-round and may have a significant negative impact on the growth of commercial algae (Cao et al. 2022). *U. lactuca* is the softest seaweed with the least sticky, elastic, crispy, cartilaginous, and cohesive properties (Figueroa et al. 2022). *Enteromorpha intestinalis* was identified as a common epiphyte on other algae and shells until it was reclassified in the genetic studies completed in the early 2000s when *E. intestinalis* was placed in the genus *Enteromorpha* (Hayden et al. 2003). It is one of the first macroalgae to colonize newly cleared surfaces on rocky shores, tidal pools, and estuaries and on the hauls of ships transport from salt to fresh water (Ibrahim & Lim 2015). *Caulerpa* spp. was found to be the most abundant green seaweed species (Farghali et al. 2022). *C. taxifolia* (Fig. 1d) is a species in the genus *Caulerpa* and it is native to the Pacific Ocean and the Caribbean Sea. It forms dense monocultures that prevent native seaweeds from establishing communities and crowding out almost all marine organisms (Paul et al. 2007). *Cladophora prolifera* (Fig. 1e) belongs to the genus *Cladophora*. It is widely distributed in the tropical and warm-temperate oceans, in both the Atlantic and Pacific Oceans, as well as the Indian Ocean. However, this species seems to be rare in the coastal area of Japan (Gestinari et al. 2010). *C. prolifera* (Fig. 1f) occurs in the Mediterranean Sea, the warm eastern Atlantic Ocean, the eastern seaboard of the United States, Mexico, and Brazil, as well as certain other scattered locations (Guiry 2021). It is one of the dominant species on the northeastern coast of California and becoming more common in distribution and occurrence over time (Aguilar-Rosas et al. 2004). *Codium* is a diverse genus of green seaweeds belonging to the *Codiaceae* family (Verbruggen et al. 2007). Some species of this genus are invasive that may disrupt the ecosystem (Meinita et al. 2022). Both *C. vermilara* (Fig. 1h) and *C. tomentosum* (Fig. 1i) belong to the genus *Codium* (Schoch et al. 2020). *C. vermilarais*, commonly referred to as *C. vermilara*, is an invasive alien species, mainly found in the Mediterranean and Atlantic Oceans. It may be easily confused with *C. fragile* (Guiry, 2021). *C. tomentosum* is native to the northeast Atlantic Ocean from the British Isles southwards to the Azores and Cape Verde. It has also been discovered around the coasts of Africa (Loiseauxde Goër & Noailles, 2008).

## Nutrient profiles

The nutritional compositions and profile of green seaweed vary among different species and depend on the growth conditions (Tables 1 and 2) (Castro-González et al. 1996; Fleurence et al. 1995; Fujiwara-Arasaki et al. 1984; Ganesan et al. 2014; Maehre et al. 2014; Manivannan et al. 2008; Matanjun et al. 2009; Pirian et al. 2018; Rasyid 2017; Ratana-Arporn & Chirapart 2006; Tabarsa et al. 2012; Yaich et al. 2011). Green seaweed is an important source of several proteins and lipids (Wong & Cheung, 2001).

**Table 1:** Nutritional compositions and their structures in different green seaweed species

Species	Nutritional composition	Structures	References
*Codium iyengarii* (Arabian Sea)	• Glycerol derivative • Derivative of transphytol • Steroid • Steroidal glycoside		Blunt et al. 2003; Blunt et al. 2004
*Cymopolia barbata* (Cuban)	• Prenylated bromohydroquinone		Blunt et al. 2004
*Pterosperma* *Cristatum* (Japanese waters)	• Carotenoid (siphonaxanthin C14:1 trans-∆^2^ ester)		
*Ulva fasciata* (Indian Coast)	• Nitrogenous glycerolipid		
*Bryopsis* sp.*,* (Oahu, Hawaii)	• Cyclic depsipeptide (Kahalalide F)		Blunt et al. 2005
*Caulerpa prolifera* (Saronicos Gulf, Greece)	• Terpene ester		
*Panicillus capitatus* (Cat Cay, Bahamas)	• Triterpene sulfate esters		Blunt et al. 2006
*Caulerpa brownii* (Tasmania, Australia)	• Diterpenoid		Blunt et al. 2007; Lahaye & Robic 2007
*Avrainvillea nigricans* (Portsmouth, Dominica)	• Glycoglycerolipid		
*Chaetomorpha basiretorsa* (Naozhou Island, China)	• Halogenated biindole • Apo-carotenone		
*Codium* *Fragile* (Qingdao coast, China)	• Clerosterol palmityl ester		
*Caulerpa taxifolia* (Nanji Island, China)	• Sequiterpene		Blunt et al. 2008
*Avrainvillea nigrans* (Portsmouth, Dominica)	• Ether-linked glycoglycerolipids		Blunt et al. 2009
*Cladophora fascicularis* (Qingdao coast, China)	• Porphyrinolactone		
*Ulva lactuca* (BoHai coast, China)	• Diastereoisomeric norisoprenoid		
*Chaetomorpha,* and *Codium*	• 28-isofucosterol		
*Tydemania* *Expeditionsis* (Herald Pass)	• Unsaturated fatty acids		Blunt et al. 2010
*Chaetomorpha basiretorsa* Setchell (Naozhou Island, China)	• Stigmasterol		
*Ulva fasciata* (Aabu-Qir, Mediterranean coast, Egypt)	• Unsaturated fatty acids		Blunt et al. 2011
Cymopolia barbata (Fairy Hill Beach, Jamaica)	• Non-halogenated cymopol		
*Bryopsis pennata* (Kahala Bay, Ohau, Hawaii).	• Cyclic depsipeptides		
*Tydemania expeditionis* (Yellow Sea, China)	• Ketosteroid		Blunt et al. 2014
*Caulerpa* *racemosa* (Zhanjiang coastline, China)	• Chloro-bisindole		
*Ulva lactuca* (Floridian Marine)	• Monounsaturated fatty acids		Blunt et al. 2015
*Caulerpa racemosa* (Zhanjiang coastline, China)	• Prenylated para-xylenes caulerprenylol		
*Caulerpa racemosa*	• Bisindole alkaloids		Blunt et al. 2016
*Caulerpa racemosa*	• Diterpenoids • α-toco pheroid		Blunt et al. 2017
*Derbesia* *marina.*	• Cyclic lipopeptides mebamamide		
*Botryococcus braunii.*	• Cyclic C33 botryococcene terpenes		Carroll et al. 2019
*Dasycaldus vermicularis*	• Sulphated coumarins		Carroll et al. 2020
*Monostroma nitidum* (Southwest coast, Japan)	• Polysaccharide		Suzuki & Terasawa 2020
*Cladophora socialis* (Fiji)	• Polyphenol (Cladophorols)		Carroll et al. 2021
*Avrainvillea longicaulis*	• Bromophenols		Carroll et al. 2022

**Table 2:** The nutrient profiles of selected edible seaweed species (% of dry weight)

Species	Protein	Ash	Dietary fiber	Carbohydrate	Lipid	References
*U. lactuca*	8.65–25	12.9–29.31	29–55	36–43	0.6–1.6	Castro-González et al., 1996; Manivannan et al. 2008; Tabarsa et al. 2012
*U. pertusa*	20–26	–	–	47	–	Fleurence et al. 1995; Fujiwara-Arasaki et al. 1984
*U.linza*	18.1	22.43	–	–	2.51	Pirian et al. 2018
*Enteromorpha intestinalis*	11.3	55.9	–	–	2.2	Pirian et al. 2018
*Cladophora*	3.42	77.8	–	–	0.88	Maehre et al. 2014
*Caulerpa lentillifer*	10.41	–	32.99	38.66	1.11	Shalaby 2011
*Caulerpa taxifolia*	12.44	–	–	23.86	0.32	Ownsworth et al. 2019
*Ulva reticulata*	21.06	–	4.84	55.77	0.75	Yaich et al. 2011
*Ulva rigida*	17.8	–	11.9	42.6	0.9	Ratana-Arporn & Chirapart 2006
*E. compressa*	17.48	31.21	2.93	44.08	3.56	Ganesan et al. 2014
*E. linza*	12.5	12.5	7.14	50.01	4.1	Ganesan et al. 2014
*E.tubulosa*	19.9	17.01	6.28	51.05	5.56	Ganesan et al. 2014

### Proteins

**Fig. 2:**
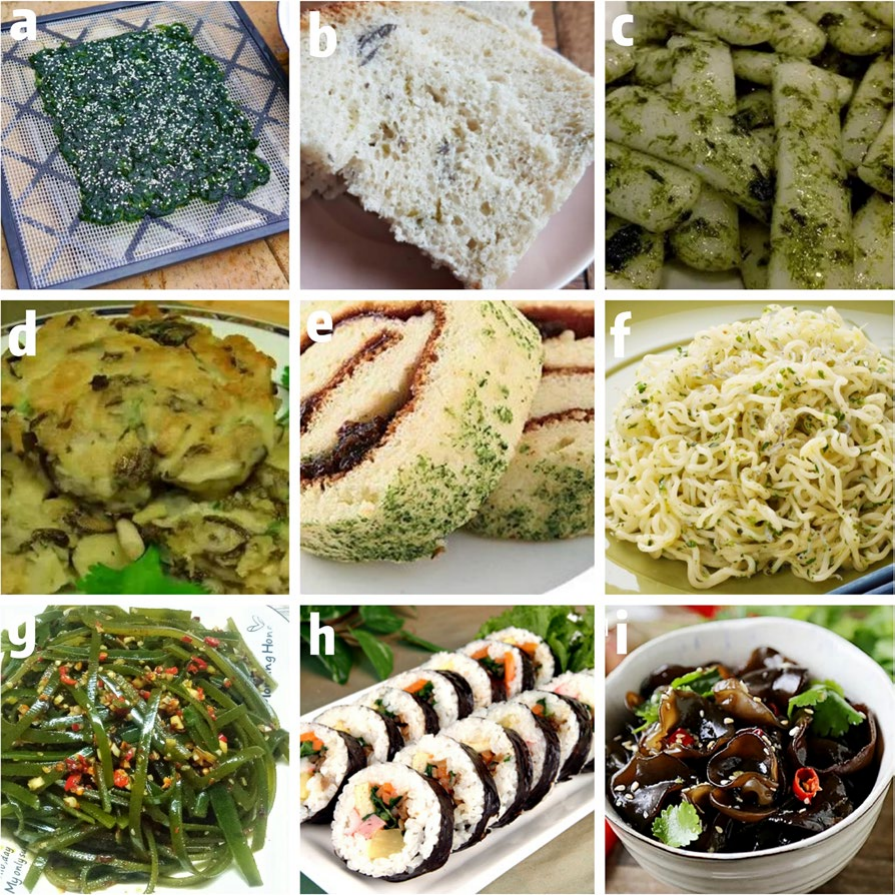
Food made with different seaweed species. **a** *Ulva lactuca;* **b** *U. intestinalis;* **c** *Enteromorpha clathrata;* **d** *E. clathrata;* **e** *U. lactuca;* **f** *E. intestinalis;* **g** *Laminaria japonica;* **h** *Pyropia;* **i** *Nostoc commune*

Protein is an essential component of the diet that animals and humans need for their survival. Macroseaweeds, especially red and green seaweeds, have attracted more attention as food sources due to their abundant protein profile and functional peptides (Harnedy & FitzGerald 2015). Seaweed is being used as human food in many Asian countries for centuries (Mišurcová et al. 2014) (Fig. 2). For example, *Cladophora* spp. is traditionally consumed as a part of the routine diet in Thailand and other Asian countries (Peerapornpisal et al. 2006; Shalaby 2011). Recently, seaweed has also made its place in North American, South American, and European dishes. Seaweed protein is a source of essential amino acids, and its amino acid profile accounts for almost half of the total amino acids (Černá 2011). Seaweed proteins also include lectins, glycoproteins, and phycobiliproteins (Echave et al. 2022). Therefore, seaweeds are considered a sustainable source of alternative nutrition (Biris-Dorhoi et al. 2020).

Green seaweed has high protein content in the dry biomass, and their protein content varies depending on the species and growing season (Benjama & Masniyom 2011). Holdt and Kraan (2011) have shown that green seaweeds of the *Ulva* genus may contain up to 44% of proteins. Among the *Ulva* genus, the edible *U. intestinalis* contains 19.5% of proteins during summer (Benjama & Masniyom 2011). Peptide is a protein derived hydrolysate, which is released from the protein structure, and exert different biological properties (Cian et al. 2012; Echave et al. 2022). Protein-derived peptides can be used as persuasive alternatives in the pharmaceutical and biotechnological industries as chemosynthetic drug candidates (Admassu et al. 2018). Kahalalide F, a cyclic peptide isolated from the green seaweed *Bryopsis* sp.*,* has potential anti-tumor activity, and it has been used in relevant clinical trials (Echave et al. 2022; Smit 2004). Lectins, a carbohydrate-binding protein, exist in many seaweed species and can interact with specific glycan structures in viruses, bacteria, fungi, and parasites (Cardozo et al. 2007; Holdt & Kraan 2011; Hori et al. 2000). Seaweed lectins have anti-inflammatory, antibiotic, and cytotoxic biological activities (Holdt & Kraan 2011; Mori et al. 2005; Zhong et al. 2020). Two lectins isolated from *C. isthmocladum* were found to inhibit the biofilm formation of *Staphylococcus aureus* and *S. epidermidis* by binding to the surface galactose. Lectins isolated from *C. cupressoides* were proven to have anti-inflammatory activity in vivo (Echave et al. 2022).

### Lipids

Lipids are basic nutrients and play an important role in maintaining human health (Holdt & Kraan 2011). Being the precursors of several signaling molecule biosynthesis such as eicosanoid, they are the biological regulators of many cellular processes. Seaweeds are known as low-energy food due to their low lipid content as compared to carbohydrate and protein content (Narayan et al. 2008). Lipid content in the commonly used seaweeds does not exceed 5% of the dry biomass. Despite low lipid content, seaweeds are rich in omega-3, and omega-6 polyunsaturated fatty acids (PUFAs) (Mišurcová et al. 2011). A previous study has shown that omega-3 PUFAs can improve the function of mitochondria (Kendel et al. 2015). The omega-3 fatty acids are precursors of various biochemical and physiological responses (Holdt & Kraan 2011). The ratio of omega-6 to omega-3 in seaweeds is in an appropriate range which is useful in preventing some chronic inflammatory diseases (Dawczynski et al. 2007; Irene et al. 2018; Shannon & Abu-Ghannam 2019). Moreover, eicosapentaenoic acid and docosahexaenoic acid can only be obtained from food and cannot be synthesized in the human body (Benjama & Masniyom 2011). Since seaweed lipids have been reported to possess high nutraceutical value, they could be potentially used in the production of low-fat foods and food products enriched with omega-3 PUFAs.

### Dietary fibers

Dietary fibers (DFs) are a group of edible carbohydrate polymers that are resistant to digestive enzymes. DFs reach the large intestine where they can be partially or fully fermented leading to the production of beneficial metabolites such as short-chain fatty acids (SCFAs) (Holdt & Kraan 2011; Makki et al. 2018). Seaweed-derived DFs have displayed multiple beneficial properties including anti-inflammatory, antioxidant, and antiviral activities, which may vary greatly due to their types and sources (Tanna & Mishra 2019). They are consisted of soluble and insoluble DFs and have been widely applied in the food, pharmaceutics, and cosmetics industries. The soluble DFs lower blood cholesterol and glucose levels by reducing the digestion and absorption of nutrients. The insoluble DFs help with constipation by increasing the volume of excreta to promote intestinal peristalsis (Benjama & Masniyom 2011). The ulvan from green seaweeds are typical soluble seaweed-derived DFs. Consumption of seaweeds rich in soluble DFs is shown to be associated with various health benefits such as reduced blood lipid levels and lower risk of some chronic diseases (Benjama & Masniyom 2011; Huang et al. 2022; Ratana-Arporn & Chirapart 2006).

### Vitamins and trace minerals

Vitamins are essential organic micronutrients, which cannot be directly synthesized by the human body and must be obtained from the diet (Wells et al. 2017). Corino et al. (2019) showed that seaweeds are a rich source of water-soluble and fat-soluble vitamins. *Ulva* contains a variety of vitamins, and regular consumption of *Ulva* can effectively prevent and treat vitamin deficiency (Kumar et al. 2008).

Minerals are inorganic substances, and the human body requires minerals for proper functioning. Trace minerals deficiency can affect different aspects of human health. The mineral content of seaweed is higher than that of land plants. In particular, seaweeds are rich in sodium, potassium, and calcium. Benjama and Masniyom (2011) analyzed the composition of two green seaweeds. The results revealed that *U. intestinalis* and *U. pertusa* have a good ratio of sodium to potassium, which contributes to body fluid balance. Magnesium is an element with catalytic function, which is significantly present in *Ulva* and *Enteromorpha* spp. (Mabeau & Fleurence 1993). Seaweeds are also a source of iron, manganese, copper, zinc, cobalt, selenium, and iodine (Corino et al. 2019; Rupérez 2002). Moreover, some of the trace elements sourced from seaweeds cannot be found in land plants (Benjama & Masniyom 2011). Taken together, green seaweeds can make an important contribution to the daily intake of minerals.

## Active substance

### Polysaccharides

**Fig. 3:**
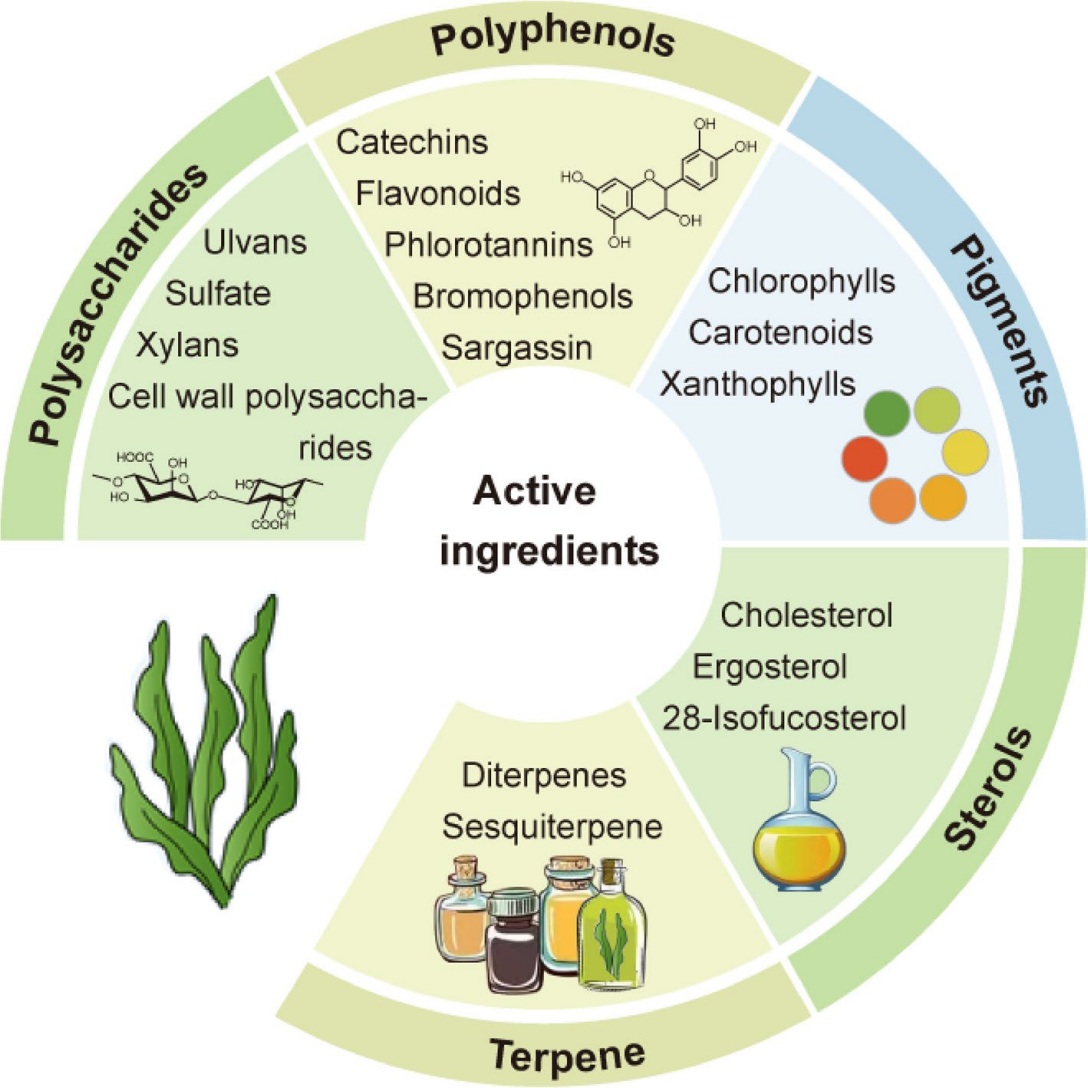
Active ingredients in green seaweeds

Seaweeds have raised great interest as an excellent source of active substances, such as complex carbohydrates and different polysaccharides (Charoensiddhi et al. 2017). The active substances in green seaweeds are depicted (Fig. 3). Polysaccharides in green seaweeds have been exploited for various applications (Alves et al. 2010). The polysaccharide content varied among different species and different parts (Charoensiddhi et al. 2017). *Ulva* spp. has a high polysaccharides content of up to 65% of dry weight (Holdt & Kraan 2011). The cell wall polysaccharides occupy various components in green seaweeds with about 38 to 54% in the total dry weight. It is reported that the *Ulva* spp. biomass contains four types of cell wall polysaccharides (Lahaye & Robic 2007).

Green seaweed primarily contains xylan and sulphated galactan (also known as ulvan) (Øverland et al. 2019; Sari-Chmayssem et al. 2019; Ulaganathan et al. 2017). Ulvans represent a sulfated polysaccharides (SPs) family extracted from green seaweeds, accounting for about 9 to 36% of the total dry biomass (Cherry et al. 2019; Morelli et al. 2017; Saravana & Chun 2017; Wells et al. 2017)*.* Ulvans are mainly comprised of sulfate, rhamnose, xylose, and glucuronic acid (Lahaye & Robic 2007). They are predicted to be directly related to a variety of biological functions (Kang et al. 2022), such as anticancer, antioxidant, antihyperlipidemic, anti-influenza, and anticoagulant activities (Abou Zeid et al. 2014; Fedorov et al. 2013; Pangestuti & Kurnianto 2017; Qi et al. 2012; Shao et al. 2013). The glycosidic linkages, molecular weight, sulfate content, and conformation of SPs can influence their bioactivities. Earlier, studies have shown that Ulvan can also be a source of rare sugar precursors for the synthesis of fine chemicals. For instance, sulfated polyaldobiuronan was used to synthesize aromatic substances, and iduronic acid plays an important role in the synthesis of heparin analogs (Duchaussoy et al. 1999; Lahaye & Robic 2007).

### Polyphenols

Studies have revealed that seaweeds contain a large concentration of antioxidant compounds such as polyphenols (Wells et al. 2017). Polyphenols are a heterogeneous group of compounds that are further categorized into phenolic acids, flavonoids, stilbenes, lignans, and other phenolic compounds based on their chemical structure. The largest proportion of phenolic compounds found in green seaweeds are bromophenols, phenolic acids, and flavonoids. Especially, the flavone glycoside hesperidin content is high up to 117 mg g^− 1^ of dry weight in some green seaweeds (Holdt & Kraan 2011). Phenolic compounds obtained from seaweeds have gained particular attention due to their specific bioactivities and health-promoting benefits (Cotas et al. 2020; Murray et al. 2018; Senthilkumar & Sudha 2012). Flavonoids, a water-ethanol extract from green seaweed *E. prolifera*, has a potential anti-diabetes effect (Yan et al. 2019). Dietary polyphenols have antioxidant activity similar to vitamins (C and E) and carotenoids (Freile-Pelegrín & Robledo 2014).

### Sterols and terpenes

Cholesterol, ergosterol, and 28-isofucosterol are the principal sterols found in green seaweeds belonging to genera *Ulva*, *Chaetomorpha*, and *Codium* (García-Poza et al. 2020; Kendel et al. 2015; Sánchez-Machado et al. 2004)*.* The cholesterol content in green seaweed species varies from 2 to 76% of total the sterol (Kendel et al. 2015). Studies have shown that plant sterols have anti-inflammatory effects and can also reduce the risk of cardiovascular disease by reducing the cholesterol level (Kendel et al. 2015; Patch et al. 2006). Terpene is the largest group of secondary metabolites in plants (Chen et al. 2011). Terpenes such as sesquiterpene and diterpenes are also known to be enriched in green seaweeds (Echave et al. 2022). Due to strong cytotoxicity, they can be used to inhibit tumors as well as bacterial growth.

### Pigments

Seaweed is a good source of natural pigments, such as chlorophylls, carotenoids, and phycobilin (Pereira et al. 2021). The color of green seaweeds is mainly due to the presence of chlorophyll a, which is contained in chloroplasts, and chlorophyll b. Except for food colorants, the pigments in seaweeds can also be used to treat patients exposed to lipophilic toxic substances. In a previous study, consumption of green seaweeds is shown to increase the excretion of dioxin in feces (Chen et al. 2017; Okai et al. 1996). Xanthophylls are oxygenated carotenoids with anti-tumor and anti-inflammatory activities (Bolhassani 2015). Fucoxanthin is a xanthophyll carotenoid, accounting for more than 10% of the total production of carotenoids in nature (Dembitsky & Maoka 2007). Fucoxanthin contains an unusual allenic bond, an epoxide functionality and a conjugated carbonyl group in the polyene chain, which makes it possess extensive biological activities, including anti-oxidation, anti-cancer, anti-inflammatory and anti-obesity (Gammone & D'Orazio 2015). What deserves to be mentioned is that fucoxanthin exhibits antioxidant properties even under anoxic conditions (Torregrosa-Crespo et al. 2018). Although brown seaweed is the main source of fucoxanthin, it also exists in green seaweeds *U. prolifera* and *C. fragile* (Li, Feng, et al. 2021). Siphonaxanthin, a keto-carotenoid has strong anti-tumor activity by reason of the special structure without epoxide or an allenic bond, and its content in green seaweeds such as *Umbraulva japonica*, *Caulerpa lentillifera*, and *C. fragile* constitutes about 0.03 to 0.1% of the dry weight (Sugawara et al. 2014; Torregrosa-Crespo et al. 2018). These health-promoting properties of pigments in seaweeds as well as its potential as a natural food colorant have led to research on the potential of pigments as a high-value nutraceutical ingredient.

## Potential biological activities

Green seaweed has the potential to meet people’s daily nutritional requirements. It also has biological activities beneficial to health and can be used in the nutraceutical and pharmaceutical industries (Table 3).

**Table 3:** Bioactive compounds in green seaweeds and their functions

Characteristics	Species	Bioactivities	References
Polysaccharides	*Monostroma angicava*	Anticoagulant property in vitro	Liu et al. 2018
	*Dictyota menstrualis*	Anti-nociceptive Anti-Inflammatory activities	Albuquerque et al. 2013
	*Enteromorpha prolifera*	Anti-oxidant and moisture absorption/retention capacities	Li et al. 2017
		Immunomodulatory	Liu et al. 2020
		Antioxidant	Li et al. 2013
Polyphenols	*Ulva lactuca*	Anti-human colorectal carcinoma	Alghazeer et al. 2008
	*Codium tomentosum*		
Pigments	*Codium fragile*	Anti-angiogenic	Ganesan et al. 2010
	*Enteromorpha prolifera*	Anti-inflammatory Antimutagenic	Okai & Hiqashi-Okai 1997
	*Caulerpa racemosa*	Antioxidant properties	Yalçın et al. 2021
Sterols	*Ulva lactuca*	Anti-breast and anticolorectal cancer agents	Arsianti et al. 2016
	*Ulva armoricana*	Anti-tumor activity of some chemotherapeutic agents	Kendel et al. 2015
Terpene	*Ulva intestinalis*	Biofertilisers	Ghaderiardakani et al. 2019

### Anti-hypertensive and anti-hyperglycaemic

Hypertension is one of the major risk factors associated with cardiovascular diseases. The inhibition of angiotensin-converting enzymes is proven to be an effective treatment approach in many clinical situations (Wijesekara & Kim 2010). Oligopeptides from *C. lentillifera* show angiotensin-converting enzyme-inhibiting properties. In addition, peptides obtained from green seaweeds *U. rigida*, *U. chlatrata*, and *U. intestinalis* also show the same effect (Syakilla et al. 2022). *C. patentiramea*, collected from the coast of Malaysia, has anti-hypertensive properties and triggers a vascular relaxant effect in the aortic rings of Wistar-Kyoto rats, which is possibly mediated by endothelium-dependent nitric oxide-cGMP pathway (Lim & Mok 2010).

While lipid is an essential nutrient for the human body, the excessive intake of lipids might lead to obesity and hyperlipidemia and increases the risk of cardiovascular disease. Diabetes is a chronic metabolic disorder characterized by high blood glucose levels, which can lead to renal dysfunction, cardiovascular disease and blindness (Hossain et al. 2020). Edible seaweed is known as low-fat food, and they are also found to reduce blood sugar levels. Pradhan et al. (2021) found that *E. prolifera* extracts can inhibit the activities of α-amylase and α-glucosidase, which lead to delayed glucose attraction in blood and plasma. Polysaccharides from other green seaweeds, such as *C. lentillifera*, *Monostroma nitidum*, and *U. lactuca,* also showed significant effects in lowering blood sugar levels (Chen, Ouyang, et al. 2022; Suzuki & Terasawa 2020; Syakilla et al. 2022).

### Antiviral activity

Rhamnose, a sulfated polysaccharide from green seaweed *Monostroma nitidum*, possesses antiviral activity against the influenza A virus by inhibiting the proliferation of envelope viruses in vivo and the adsorption and entry of viruses in vitro (Terasawa et al. 2020). SPs in *Ulvans* and *Caulerpa* genera also exert the same effect. In addition, caulerpin an alkaloid has a high binding ability to SARS-CoV-2 protein receptors, which enhances its antiviral ability (Shah et al. 2020). Polysaccharides from *Monostroma latissimum* can target capsid protein VP1 and inhibit viral replication before or during the adsorption of the virus. After adsorption, the early infection may also be prevented by regulating EGFR/PI3K/AKT signaling pathway. In addition, it significantly increased the survival rate of 71 mice infected with enterovirus and decreased the viral titers (Wang et al. 2018). Lopes et al. (2017) evaluated the ability of seven chemically modified polysaccharides from *Enteromorpha compressa* to prevent the herpes simplex virus (HSV) infection. The polysaccharide derivative, SU1F1 showed antiviral activity and played an inhibitory role at the early stage of HSV replication.

### Anti-cancer and immunomodulatory activities

Ahmed and Ahmed (2014) generated an Ehrlich ascites carcinoma-bearing mice model in vivo. After the *Ulvan* polysaccharides treatment, the number of dead cells in Ehrlich ascites carcinoma-bearing mice significantly increased. In addition, the protein expression of proapoptotic mediator p53 increased, whereas the expression of anti-apoptotic protein Bcl2 decreased. *Ulvan* polysaccharides were suggested to play anti-tumor roles by inducing apoptosis and inhibiting cell division. In addition, Zhao et al. (2020) found that *U. lactuca* polysaccharide (ULP) could inhibit the proliferation of tumor cells and also had an immunomodulatory effect. Glucuronic acid sulfate extracted from *Capsosiphon fulvescens* induces apoptosis in HT-29 cancer cells by activating a protease-dependent apoptotic pathway (Choi et al. 2019). In rats, ULP prevented 7,12-dimethylbenzoanthracene-induced carcinogenic diseases after 10 weeks of treatment by promoting cell apoptosis and inhibiting oxidative stress as well as inflammatory response (Abd-Ellatef et al. 2017). Other components such as polyphenols and flavonoids extracted from *U. lactuca* and *C. tomentosum* also exhibited anti-tumor activity and inhibited the growth of Caco2 cells (Alghazeer et al. 2008). Moreover, the crude extracts of green seaweeds also displayed anticancer activity. Further, glycolipids from *U. armoricana* showed promising anti-proliferative activities on non-small cell lung cancer (NSCLC-N6) cells (Kendel et al. 2015).

Sulfated polysaccharide from *E. prolifera* stimulates macrophages to release a large amount of nitric oxide in RAW 264.7 cells and induces the expression of multiple cytokines, which play an important role in regulating various immune responses (Kim et al. 2011). EP2, a water-soluble polysaccharide extracted from *E. prolifera* plays an immunomodulatory role in cyclophosphamide-induced immunosuppressed mice (Liu et al. 2020). The polysaccharide extracted from *C. lentillifera* enhanced the activity of RAW 264.7 macrophages and stimulated the production of nitric oxide in mice (Sun et al. 2018). The fractionated sulphated polysaccharides isolated from *U. intestinalis* showed stronger immunomodulatory activity by stimulating the production of pro-inflammatory cytokines in macrophage J774A.1 cells. These results indicated that *U. intestinalis* polysaccharides could be used as potential immunomodulators (Peasura et al. 2016).

### Anticoagulant activity

SPs from seaweeds have been widely used as a substitute for heparin, due to its effective anticoagulant activity. The anticoagulant activity of SPs from green seaweed is significantly higher than those from red and brown seaweeds (Wang et al. 2014). Shanmugam et al. (2002) studied the anticoagulant activity of *Codium* collected from the Indian coast, and the results showed that several *Codium* species could produce SPs with strong anticoagulant activity. Athukorala et al. (2007) depicted that the hot water extract of *C. fragile* SPs shows a prolonged activated partial thromboplastin time. Sulfated ulvan isolated from the *U. rigida* showed stronger anticoagulant activity as compared to commercial anticoagulants (Adrien et al. 2019).

## Applications of green seaweeds

**Fig. 4:**
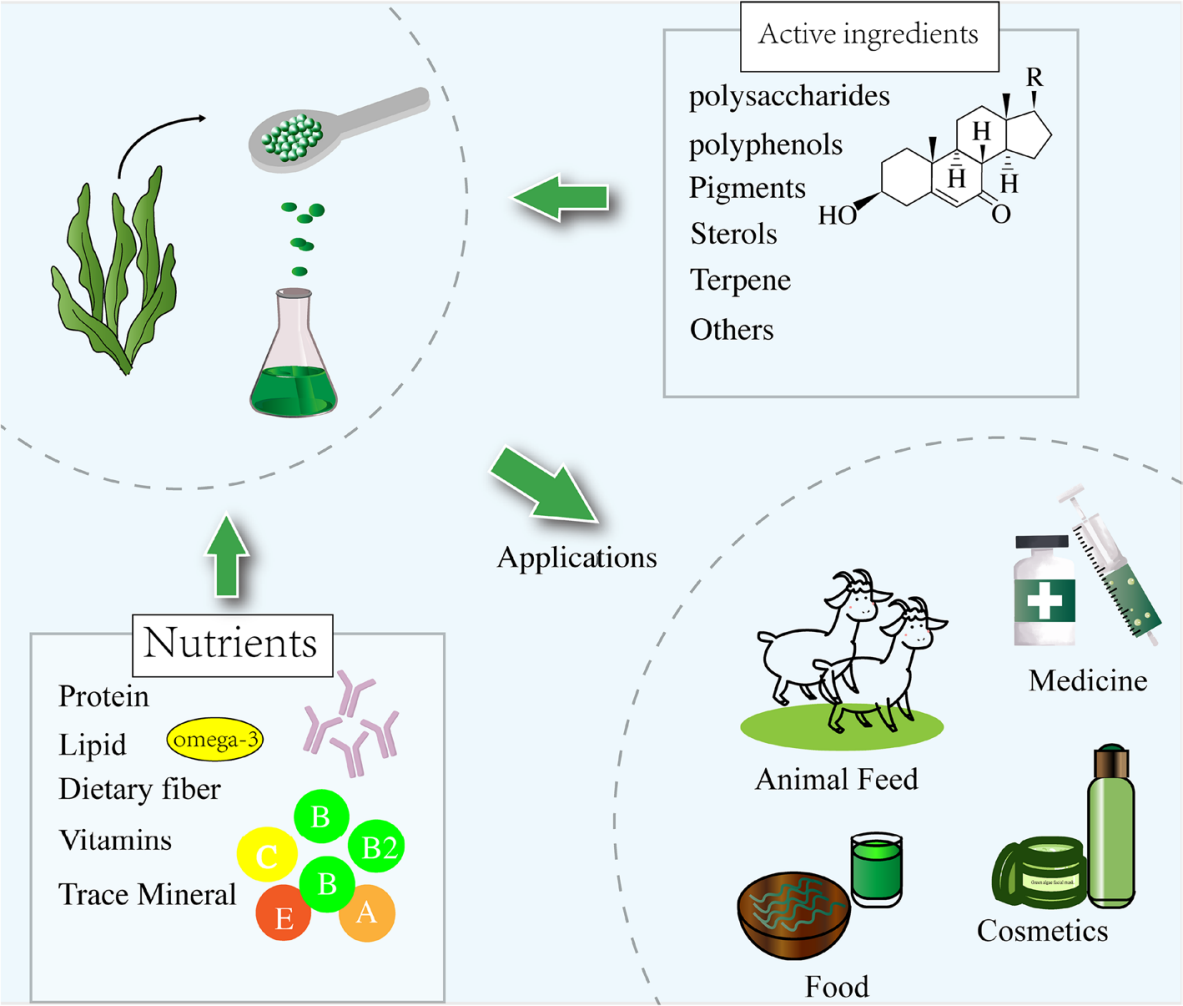
Applications of green seaweeds

Seaweed is considered a health-promoting substance, due to its nutritional composition and bioactive ingredients, especially those which are not present in terrestrial food sources. Therefore, seaweeds are widely used in food, medical treatment, cosmetics, feed, and other fields (Fig. 4).

### Food

The consumption of seaweed has a long dietary history. Not only Asian countries but also western countries began to consume seaweed in the form of both fresh and processed food products (Leandro et al. 2020; Løvdal et al. 2021). For instance, nori, wakame, and kombu are used as either raw materials or flavoring agents for other food items. These seaweeds are considered to be the most ideal dietary sources (Zhang et al. 2022). Grinding seaweed into powder and adding it to the bakery or starchy foods can improve the nutritional content of food and also enhance the flavor (Prabhasankar et al. 2009; Quitral et al. 2022). In addition, seaweed can provide physicochemical and textural characteristics when contained as food ingredient, such as SPs present in ulvan can improve the structure and strength of food products (Quitral et al., 2022). Eating foods with a high proportion of DF enhances the sense of satiety, reduces postprandial blood glucose, cholesterol, and islet levels, and effectively alleviates constipation (Hall et al. 2012). World Health Organization (WHO) recommends the ingestion of 25–35 g/day of DF (Reynolds et al. 2019). DF content in *U. rigida* is as high as 38 to 41% of the dry weight (Pereira 2011). Seaweeds are also rich in minerals and trace elements. The iodine content in green seaweeds is lower than brown seaweeds but higher than land plants, with up to 100–300 ppm (parts per million) of dry weight, which can easily meet the daily nutritional requirement for adults (Schultes 1988). Several seaweeds from the Norwegian coast were characterized by Irene et al. (2018). The findings suggested that *U. intestinalis* possessed the highest Fe content (5800 mg/kg dry weight). Some trace elements are lacking or present in low concentrations in land vegetables, which makes seaweeds an important source of minerals in the regular diet. A recent study has also shown that seaweed can reduce the risk of allergic reactions caused by ingesting food proteins (Yu et al. 2020). Therefore, seaweeds are preferred as functional food and nutraceuticals to improve human health.

Seaweeds are considered healthy foods with high nutritional value and can fight chronic diseases and nutritional deficiencies. However, the nutrients in seaweed-derived products may be lost due to the inevitable processing, as well as due to digestion, distribution, and low bioavailability during the food-to-organism cycle. In addition, seaweeds easily accumulate pollutants, metals, and other toxic substances (Henriques et al. 2017; Ownsworth et al. 2019). Studies have shown that the accumulation of inorganic arsenic increases the incidences of cancer and cardiovascular diseases and also has an impact on the nervous system (Arslan et al. 2017). Park et al. (2015) reported a foodborne intoxication caused by the ingestion of green seaweeds. Therefore, it is necessary to develop seaweed-derived food with high added value (Bleakley & Hayes 2017) by detecting and limiting the concentration of toxic substances and enhancing the consumption of beneficial ingredients to meet health requirements.

### Animal feed

It has been verified that seaweed can be used as a supplementary source of proteins, polysaccharides, dietary lipids, etc. (Holdt & Kraan 2011). It contains several health-promoting ingredients, which have anti-bacterial, anti-inflammatory, and other bioactive properties. Therefore, seaweed powder is also added to the animal feed. Its efficacy varies greatly among different seaweeds. In a previous study, *Ulva* powder was fed to Nile tilapia as a replacement for dietary lipids. The physiological property of Nile tilapia and the feed utilization capacity were improved (Ergün et al. 2009). Feeding *Ulva* showed a beneficial effect on the nutritional value of sea bream (Mustafa et al. 1995). It is to be noted that while some minerals and metals in seaweeds are beneficial, a few toxic substances such as lead, copper, and mercury are also present. It is reported that seaweeds absorb arsenic (AS) in the marine environment. Livestock feed containing toxic AS results in the accumulation of AS in the body which leads to mental damage. The AS content in green seaweeds is lower than that in brown seaweeds (Francesconi & Edmonds 1996). Bonanno et al. (2020) found a massive accumulation of trace metals in *U. lactuca* from the Mediterranean Sea. Therefore, while adding seaweed to animal feed, it is necessary to detect harmful components. This is a huge challenge faced in the aquaculture industry. Adding seaweed to feed can improve various growth indexes of aquatic and terrestrial animals, reduce disease frequency, replace antibiotics and other drugs, and lower the residue of veterinary drugs (Bizzaro et al. 2022).

### Medicine

China has a long history of using seaweed in traditional medicine. Green seaweeds, including *U. conglobata*, *U. lactuca, Enteromorpha* sp.*,* and *Codium* sp. have been widely used in herbal medicine. These species are used to treat goiter, cough, bronchitis, tonsillitis urinary diseases, and dropsy. Also, they are known to have heat-clearing and detoxification effects (Chengkui et al. 1984). Chen, Wu, et al. (2022) found that *U. lactuca* oligosaccharide can be exploited further to design an effective therapy for restoring blood glucose metabolism in elderly patients with type 2 diabetes. Polysaccharides extracted from *U. lactuca* have been recognized to possess potential anti-tumor, anti-oxidant, anti-hyperlipidemia, and anti-diabetic properties (Chen, Ouyang, et al. 2022). Ouyang et al. (2022) found that *Enteromorpha prolifera* oligosaccharide regulates glucose metabolism in elderly diabetic mice through the gut-brain axis, indicating that *E. prolifera* oligosaccharide is a new natural drug for treating elderly diabetes. The anti-hyperuricemic effects of *E. prolifera and U. lactuca* polysaccharides were also reported (Li, Chen, et al. 2021; Li, Gao, et al. 2021). Altogether, these studies indicate that green seaweeds possess potential medicinal properties.

### Cosmetics

Seaweed, as a natural, safe, efficient, and sustainable marine resource has attracted more attention in the past few years. They are used in cosmetics as ingredients, additives, and active agents, and can provide texture-related characteristics such as thickening, emulsification, and wetting (López-Hortas et al. 2021). Ulvans have anti-aging and anti-herpesvirus properties (Fournière et al. 2021; Lahaye & Robic 2007). A hydrolyzed extract Aosaine® obtained from *U. lactuca* possesses anti-aging, anti-wrinkle, and collagen-stimulating properties. PUFAs can benefit skin barrier protection. Vitamins are essential for many functions related to human skin. Green seaweeds also contain higher levels of the vitamin B complex. In particular, niacinamide, nicotinic acid, and nicotinate esters present in green seaweeds are active forms of vitamin B3, which are added to skincare products. The mineral proportions in seaweed are close to human skin, therefore, it can be absorbed easily (Kim 2014). This high affinity makes seaweed-derived skin care products and cosmetics more desirable due to several beneficial effects on the skin (moisturizing, promoting metabolism, reducing facial acne, and slowing down aging). High concentrations of minerals and trace elements can also help in providing protection against ultraviolet rays (Alves et al. 2010; Guillerme et al. 2017). Carotenoids and phycobilins present in seaweeds can be used as natural color enhancers and also have hypoallergenic characteristics (Couteau & Coiffard 2020). Terpenoids and sulfur compounds impart a special aroma to cosmetics and effectively improve the sensorial properties of products (Cunha et al. 2015). Extracts from green seaweeds proved effective treatment against acne vulgaris and oral bacteria (López-Hortas et al. 2021). The glucuronic acid extracted from *Codium tomentosum* showed an ability to regulate skin moisture distribution (Kim et al. 2008).

## Prospects, challenges, and future aspects

Investigations are underway on different green seaweed species owing to their potential use in nutraceutical, pharmaceutical, cosmeceutical, and animal feed. Research focusing on the exploration of active substances extracted from green seaweeds is conducive to the discovery of new active compounds which improves the utilization value of marine resources. However, the functional properties of green seaweeds are still underexplored, and their bioactive compounds need to be characterized further for their efficient utilization.

Green seaweeds contain a variety of polysaccharides, vitamins, proteins, organic acids, and other bioactive substances. Polysaccharides are the most important compounds, which have gradually become a research hotspot in the fields of functional food and medicine. However, there are still many problems that need to be addressed. In particular, the specific mechanism underlying the biological activity and the structure-activity relationship of polysaccharides need to be investigated further. It is imperative to study its advanced structures such as spatial conformation to provide a theoretical basis for revealing the structure-activity relationship of polysaccharides. Green seaweeds as the main ingredient of animal feed can exert positive effects on animal growth and meat quality, as well as environmental sustainability. They are promising alternatives to staple food crops such as feed and forage. However, feeding seaweeds are also associated with potential constraints, which include excessive bioaccumulation of inorganic elements such as heavy metals. The importance of seaweed is not only shown in functional food, animal feed, and drugs, but also biofuels and biofertilizers. Fatty acids in seaweeds have potential application prospects in generating biofuels. If the cost of seaweed biodiesel is effectively reduced, it will not only alleviate the shortage of fuel resources greatly, but also reduce the emission of greenhouse gases such as carbon dioxide and acid gases such as sulfides.

## Conclusions

Several substantial evidence are there to extrapolate green seaweeds as functional foods with high nutritive value. The nutritional composition and bioactivity of green seaweeds were reviewed in this study. The findings revealed that green seaweeds are rich in polysaccharides, proteins, PUFAs, minerals, and bioactive compounds, which contribute to several health benefits, such as cardio-protective, antibacterial, anti-tumor, anti-inflammatory, anti-oxidative and immunostimulatory properties. Seaweed-derived food products are preferred by many bodybuilders due to their low-calorie and high-fiber characteristics. Also, they contain a variety of minerals and vitamins. Further, the potential use of green seaweeds in medical treatment and disease prevention makes them an excellent target to find and develop new drugs. However, green seaweeds are still underutilized, and extensive studies on different isolates and extracts from green seaweeds are extremely important. In addition, further understanding of their bioactivity and mechanisms of action is also needed for their efficient utilization. Besides, they also have great prospects in biofuels and biofertilizers. The development and utilization of seaweed is a very promising direction, and extensive in-depth research is required to exploit the maximum potential of green seaweeds.

## Data Availability

The datasets used and/or analyzed during the current study are available from the corresponding author on reasonable request.

## Abbreviations

DFs: Dietary fibers
HSV: Herpes simplex virus
PUFAs: Polyunsaturated fatty acids
SCFAs: Short-chain fatty acids
SPs: Sulfated polysaccharides
ULP: *Ulva lactuca* polysaccharide
WHO: World Health Organization
